# Case Report: Prenatal diagnosis of gastrointestinal defects and immunodeficiency syndrome caused by compound heterozygous mutations in TTC7A gene

**DOI:** 10.3389/fimmu.2025.1611155

**Published:** 2025-08-18

**Authors:** Shuning Han, Miaomiao Wang, Pengzhen Jin, Jiawei Hong, Chunfei Xu, Minyue Dong

**Affiliations:** ^1^ Women’s Hospital, School of Medicine, Zhejiang University, Hangzhou, China; ^2^ Key Laboratory of Reproductive Genetics (Zhejiang University), Ministry of Education, Hangzhou, China

**Keywords:** gastrointestinal defect and immunodeficiency, TTC7A, immune deficiency, enteropathy, whole exome sequencing, prenatal diagnosis

## Abstract

Gastrointestinal defects and immunodeficiency syndrome (GIDID) is a rare and complex disorder characterized by concurrent dysfunction of the digestive and immune systems. Typically manifesting in infancy or early childhood, GIDID carries a severe prognosis with high early mortality rates. The syndrome has been specifically linked to mutations in the TTC7A gene located on chromosome 2p21. Although GIDID can present during the fetal period, reports of prenatal diagnosis remain exceptionally rare. In this study, we investigated a case involving a fetus with gastrointestinal abnormalities detected during prenatal screening, conceived by a consanguineous couple. Following termination of the pregnancy, whole-exome sequencing of the affected fetus revealed compound heterozygous variants (c.2378del and c.2357G>T) in the TTC7A gene (OMIM:609332). These findings provide critical insights for the prenatal diagnosis of GIDID and enhance fetal detection rate. Furthermore, this study expands the spectrum of known pathogenic mutations in the TTC7A gene and underscores the significant utility of fetal whole-exome sequencing for diagnosing this condition.

## 1 Introduction

Gastrointestinal Defects and Immunodeficiency Syndrome is a rare genetic disorder characterized by abnormalities in both the gastrointestinal system and the immune system, which presents clinically as immunodeficiency, very early onset inflammatory bowel disease (VEOIBD) and/or multiple intestinal atresia (MIA) (1–3). Individuals with this condition are prone to premature birth and face an extremely severe prognosis with high early mortality. Notably, over half die before their first year of life (3–6).

GIDID is inherited in an autosomal recessive pattern, with molecular genetic studies establishing its association with biallelic mutations in the TTC7A gene located at chromosome 2p21 (4, 7). The TTC7A gene product, a critical regulatory protein, is essential for the proper development and homeostasis of both intestinal epithelial cells and lymphocytes. Molecular analyses have demonstrated that loss-of-function mutations within the tetratricopeptide repeat (TPR) domains disrupt critical protein-protein interactions and transcriptional regulation processes (1, 8, 9). At the cellular level, TTC7A deficiency manifests through distinct pathogenic mechanisms in different cell types. In enterocytes, the impaired regulatory control over the RhoA signaling pathway results in profound disruptions of apicobasal polarity (2). Concurrently, in the lymphoid compartment, thymic T cells exhibit defective proliferation, impaired adhesion capacity, and aberrant migratory patterns (10). These cellular disturbances collectively contribute to the characteristic pathological features of GIDID, including progressive apoptotic enteropathy and profound lymphocyte depletion, ultimately leading to the reversal of cellular polarity.

The prenatal manifestations of GIDID remain poorly characterized in current literature. In a seminal study conducted by Amelie Busolin’s research team, comprehensive antenatal findings were documented in 30 affected patients. The most prevalent ultrasonographic features included intestinal dilatation/atresia (n=19), hydramnios/polyhydramnios (n=14), and intraluminal calcifications (n=10) (6). Given the profound implications for clinical management, the establishment of a definitive genetic diagnosis through prenatal testing is essential for informed therapeutic decisions. Current guidelines recommend the implementation of whole-exome sequencing (WES) as a first-tier diagnostic approach when severe fetal structural abnormalities are detected, particularly those involving the gastrointestinal tract.

In this study, we present a prenatal case of gastrointestinal defects and dysplasia in a fetus from a non-consanguineous couple. Comprehensive prenatal evaluation through whole-exome sequencing revealed compound heterozygous mutations (c.2378del and c.2357G>T) in the TTC7A gene, both classified as pathogenic variants according to ACMG guidelines. This molecular confirmation enabled the definitive prenatal diagnosis of GIDID. While the prenatal presentation of GIDID remains poorly documented in the literature, our findings contribute to the expanding knowledge of its fetal manifestations. This investigation not only broadens the mutational spectrum of the TTC7A gene but also demonstrates the diagnostic efficacy of integrating advanced ultrasonography with fetal whole-exome sequencing for prenatal identification of GIDID.

## 2 Materials and methods

### 2.1 Case presentation

**Figure 1:**
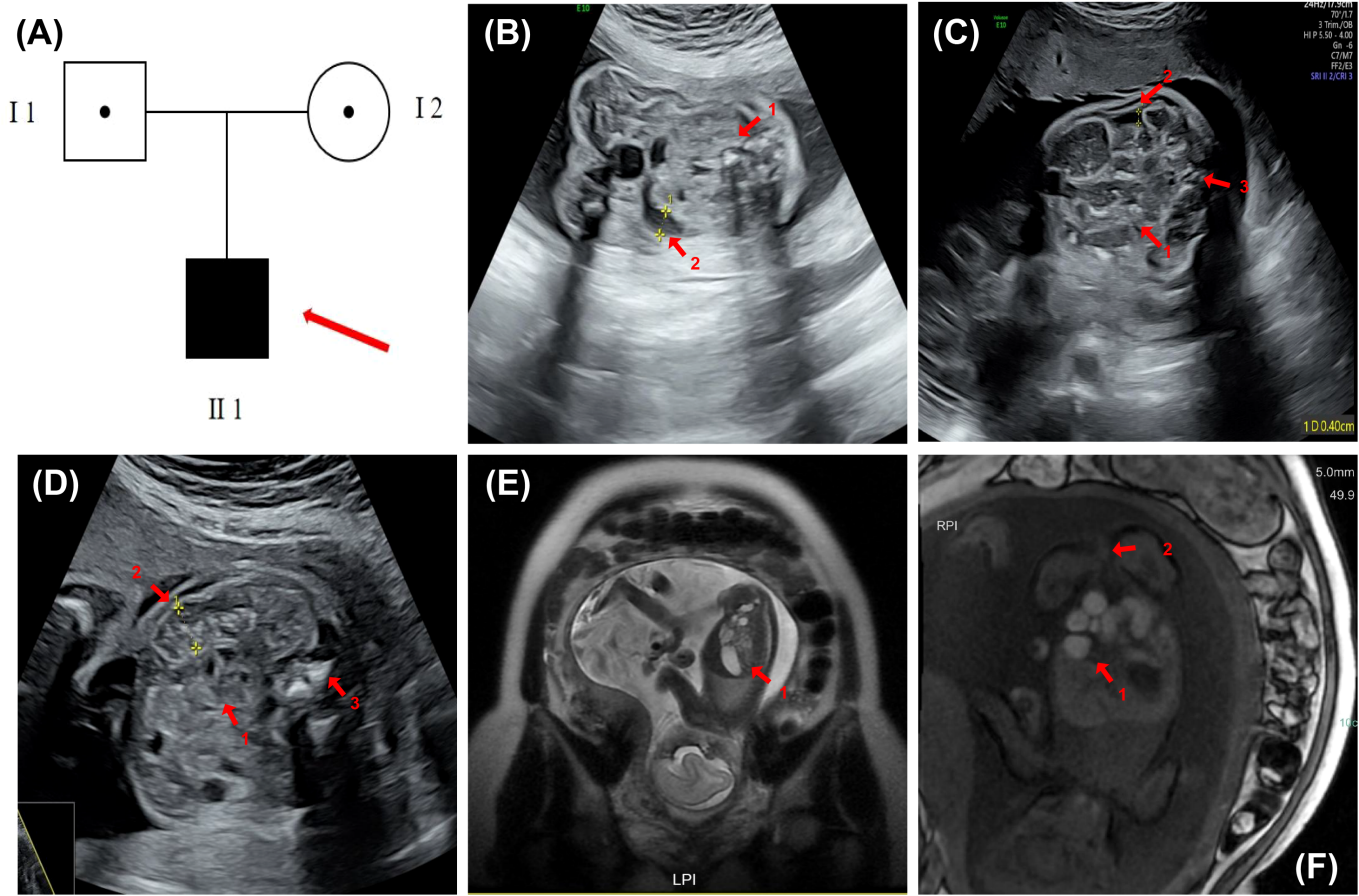
Pedigree and prenatal imaging findings. **(A)** Family pedigree. The solid square denotes the affected male fetus. **(B)** Fetal ultrasound at 24 weeks of gestation demonstrating localized bowel hyperechogenicity, approaching bone echogenicity (indicated by arrow 1), segmental bowel dilation with a maximum internal diameter: 0.8 cm (indicated by arrow 2), and mild polyhydramnios (II-1). **(C)** Fetal ultrasound at 30 weeks gestation revealing localized bowel dilation (maximum internal diameter: 0.8 cm, second arrow) with reduced internal echogenicity and sound transmission (first arrow), accompanied by meconium peritonitis and an anorectal target sign (third arrow). The amniotic fluid is slightly increased (II 1). **(D)** Fetal ultrasound at 26 weeks of gestation showing mild intestinal echogenicity (approaching bone), multiple scattered hyperechoic foci (arrow 1), localized distal dilation (max diameter ~1.0 cm, arrow 2), and an anal “target sign” (arrow 3) (II 1). **(E)** Fetal abdominal MRI at 24 weeks showed mild dilatation of the ascending and transverse colon (maximum width ~8.6 mm), increased fluid and reduced meconium in the descending and sigmoid colon (maximum width ~7.2 mm), and a slender rectum (arrow 1). **(F)** Fetal abdominal MRI at 30 weeks revealed localized intestinal dilatation (maximum diameter ~2.1 cm) with poor sound transmission and multiple scattered hyperechoic foci; a 0.4 cm-wide interintestinal fluid collection (arrow 1); and a visible anal “target sign” (arrow 2).

A 31-year-old healthy primigravida, with no history of consanguinity and an unremarkable medical and family history, was referred to our prenatal diagnostic center following the detection of intrauterine gastrointestinal abnormalities in the fetus (**Figure 1A**). First-trimester combined screening revealed a decreased level of pregnancy-associated plasma protein A (PAPP-A) at 0.33 multiples of the median (MoM). Both mid-trimester serum screening and non-invasive prenatal testing (NIPT) in the second trimester indicated a low risk for common chromosomal aneuploidies. Ultrasonographic evaluation at 30 weeks of gestation demonstrated localized intestinal dilation with a maximum internal diameter of 0.8 cm, accompanied by features of meconium peritonitis, a characteristic ‘target sign’ at the anal region, and polyhydramnios. Fetal magnetic resonance imaging (MRI) at 30 weeks further confirmed gastrointestinal abnormalities, including rectal atresia, multiple suspected colonic strictures, and persistent polyhydramnios.

Following a comprehensive diagnostic evaluation, encompassing fetal genetic testing, MRI, and extensive genetic counseling, the pregnancy was medically terminated at 31 weeks of gestation. The utilization of clinical data and medical records was conducted in accordance with ethical standards, having obtained approval from the Institutional Review Board (IRB) of the Women’s Hospital, School of Medicine, Zhejiang University. This study adhered to the ethical principles outlined in the Declaration of Helsinki. Written informed consent was obtained from all participants prior to their inclusion in the study.

### 2.2 Whole exome sequencing

DNA samples obtained from the fetus and the parents were subjected to whole exome sequencing using the Illumina HiSeq2000 platform (Illumina, San Diego, CA, USA), following the manufacturer’s standardized protocols. Sequencing achieved a minimum coverage depth of 20-fold for over 98.98% of the exonic regions. Identified variants were annotated and filtered against population databases, including the International Genome Sample Resource (1000 Genomes, http://www.internationalgenome.org), the Genome Aggregation Database (gnomAD, http://gnomad.broadinstitute.org/), and the Exome Aggregation Consortium (ExAC, http://exac.broadinstitute.org/). To assess the potential pathogenicity of the variants, computational prediction tools were employed, including SIFT (http://sift.jcvi.org/), SPIDEX (http://tools.genes.toronto.edu/), MaxEntScan (http://hollywood.mit.edu/), and MutationTaster (http://www.mutationtaster.org/). Variant interpretation was conducted in accordance with the guidelines established by the American College of Medical Genetics and Genomics (ACMG). Only variants deemed clinically relevant and consistent with the patient’s phenotypic manifestations were reported.

### 2.3 Sanger sequencing

Sanger sequencing was carried out to validate the variants with an ABI 3130 DNA analyzer (Applied Biosystems™). Given the exceedingly narrow spacing between the two mutation sites, it was unfeasible to design two distinct pairs of primers. Consequently, a single pair of primers was carefully designed to simultaneously recognize both of these sites. The forward primer (5′-TAACAGTGCCCAAGCCAGAG-3′) and reverse primer (5′-CATGGGTGAGGGTGAAGGAC-3′) were used to amplify the PCR products in the TTC7A gene. The procedure of the PCR was as follows: 95°C for 10 min, then followed by 35 cycles of 60°C for 30 s and 72°C for 1 min, and then 72°C for 10 min. The reaction was kept at 16°C.

## 3 Results

### 3.1 Prenatal imaging

The patient underwent regular prenatal examinations, and structural abnormalities were first detected at 23 weeks of gestation during a routine obstetric 4D ultrasound, which indicated possible gastrointestinal anomalies. Subsequently, more detailed ultrasound scans and fetal abdominal MRI examinations were performed at 24 to 30 weeks of gestation, respectively.

At 24 weeks, the ultrasound revealed localized echogenicity enhancement in the fetal bowel, approaching the echogenicity of bone, along with localized bowel distension. The widest internal diameter measured approximately 0.8 cm, and there was a slight increase in amniotic fluid (**Figure 1B**). The fetal MRI performed at 24 weeks of gestation showed mild dilation of the colon, with a maximum diameter of approximately 0.86 mm. The colonic contents appeared predominantly fluid with minimal meconium, and the rectum was thin. Distal colonic stenosis was suspected based on these findings (**Figure 1E**).

At 26 weeks of gestation, the ultrasound demonstrated mildly increased echogenicity of the fetal bowel, with some areas approaching bone-like echogenicity. The proximal bowel was moderately dilated (up to 0.4 cm), and the distal segment was slightly enlarged (up to 1.0 cm) with poor acoustic transmission and scattered hyperechoic foci. A “target sign” was also observed in the anal region (**Figure 1D**).

By 30 weeks, the ultrasound showed persistent localized fetal bowel dilation with the widest internal diameter remaining at approximately 0.8 cm, accompanied by poor internal sound transmission, meconium peritonitis, and an “anorectal target sign.” The amniotic fluid was again slightly increased (**Figure 1C**). At 30 weeks of gestation, an MRI examination was conducted on the fetus. The results indicated abnormalities in the fetal digestive tract, suggesting possible rectal atresia and multiple colonic strictures, along with polyhydramnios (**Figure 1F**).

Based on the findings from ultrasound and MRI, the fetus primarily presented with gastrointestinal malformations and polyhydramnios. From 24 to 30 weeks of gestation, the abnormalities progressively worsened. The colon became increasingly dilated, eventually developing multiple areas of stenosis, while the rectum progressed from being slender to showing signs of complete atresia.

### 3.2 Identification of compound heterozygous variants

Compound heterozygous variants c.2378del and c.2357G>T were identified in the TTC7A gene (OMIM:609332). Both variants are located in the last exon of the gene (exon 20) and were classified as “variants of uncertain significance” according to the ACMG guidelines (2015). The c.2378del variant, located in the last exon (exon 20), is predicted not to undergo nonsense-mediated decay (NMD) but affects a critical protein functional domain (PSV1). This variant has not been reported in the reference populations of the 1000 Genomes, the China Genome Database, the Exome Aggregation Consortium, or the Genome Aggregation Database (PM2). It is maternally inherited and results from a frameshift mutation due to the deletion of the 2378th base in the gene segment. The c.2357G>T variant is predicted to have a deleterious effect on the gene or gene product, as indicated by a REVEL score of 0.893 (PP3). This variant has also not been found in the aforementioned reference populations (PM2). It is paternally inherited and results from a missense mutation where the G base at position 2357 is substituted with a T base.

### 3.3 Confirmation of the candidate mutations

**Figure 2:**
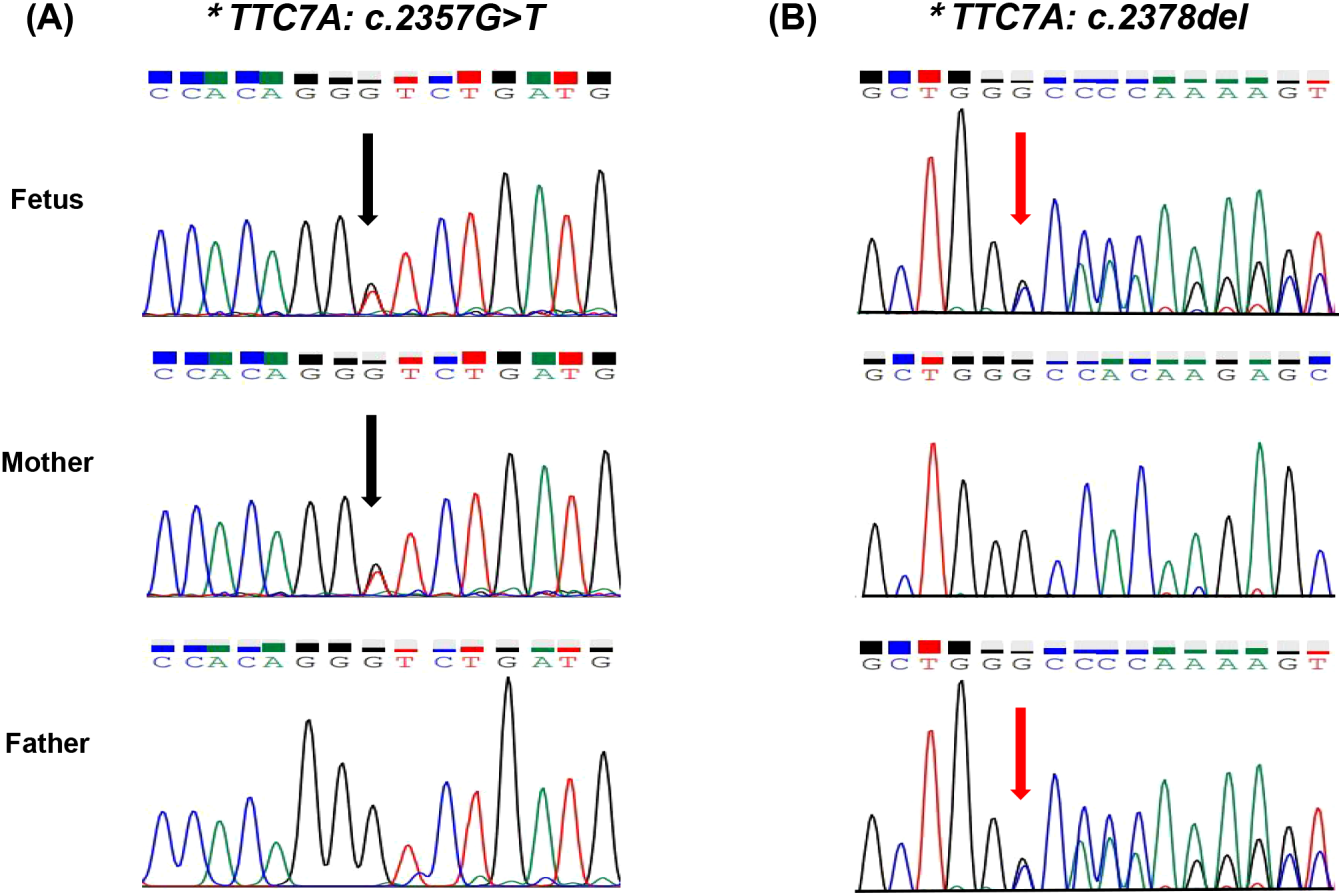
**(A)** Sanger sequencing of the compound heterozygous variants in the TTC7A gene. Sanger sequencing reveals the variant c.2357G>T (indicated by the black arrow). **(B)** Sanger sequencing reveals the variant c.2378del. The fetus carries compound heterozygous variants, with c.2357G>T inherited from the mother and c.2378del inherited from the father.

One primer set was designed, and Sanger sequencing was performed on the fetus, father and mother. As shown in **Figure 2**, the mutations of c.2357G>T and c.2378del were confirmed in the fetus (II-1), which were inherited from his mother (I 2) and father (I 1), respectively.

### 3.4 Protein structure prediction

**Figure 3:**
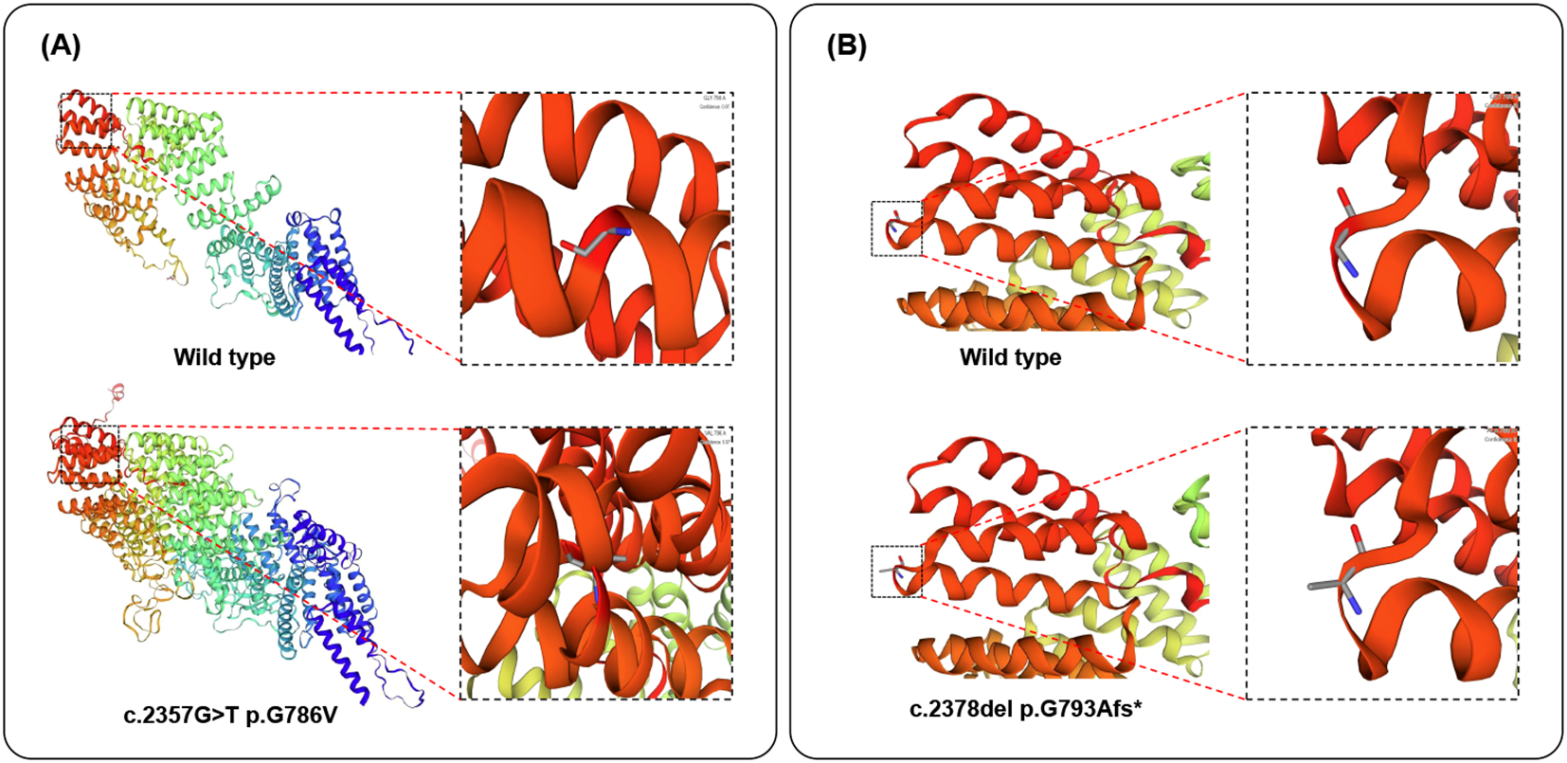
Structure of the TTC7A protein. **(A, B)**. The structure of wild-type, c.2357G>T missense mutation of TTC7A proteins, and c.2378del frameshift mutation of TTC7A proteins as predicted by the Swiss Model respectively.

An analysis of missense variant and frameshift variant was performed by homology modeling in DeepView/Swiss-PdbViewer using the most similar structures available in the Protein Data Bank. Protein structure 3D modeling was performed using Swiss-PdbViewer 4.1.0 (Swiss Institute of Bioinformatics, https://swissmodel.expasy.org/). The primary sequence of each candidate protein was loaded in Swiss-PdbViewer and aligned onto suitable modeling templates retrieved from SWISS-MODEL. The wild-type and c.2357G>T missense mutation Tetratricopeptide repeat protein 7A is shown in **Figure 3A**. The wild-type and c.2378del frameshift mutation Tetratricopeptide repeat protein 7A were superposed in three-dimensional (3D) space using Swiss-PdbViewer 4.1.0 (**Figure 3B**). The TTC7A c.2357G>T missense mutation not only replaces glycine at position 786 of the peptide chain with valine but also alters the protein structure, resulting in an increase in helices and tighter helical packing, which may affect protein function. While the TTC7A c.2378del frameshift mutation replaces glycine at position 793 of the peptide chain with alanine, without causing significant structural changes in the protein, potentially impairing protein function.

## 4 Discussion

GIDID is a rare and severe clinical disorder first documented in 1974 (11). The condition was initially characterized by the presence of multiple intestinal atresias in patients from consanguineous families (12). Clinically, GIDID manifests as multiple intestinal atresias accompanied by varying degrees of severe immunodeficiency or sepsis. Additionally, some patients develop inflammatory changes in the small intestine or colon, or experience recurrent intestinal strictures (13–15). In a comprehensive classification by Amelie Busolin and colleagues, three distinct phenotypic presentations were identified based on the triad of primary clinical features (multiple intestinal atresia, immune deficiency, and enteropathy): (1) immune deficiency with multiple intestinal atresia without enteropathy (ID/MI), (2) immune deficiency with enteropathy without atresia (ID/E), and (3) immune deficiency with both multiple intestinal atresia and enteropathy (ID/MIA/E) (4). The prognosis of GIDID remains generally poor, with most cases presenting severe, early-onset symptoms that contribute to high mortality rates within the first year of life. Amelie Busolin et al. reported a mortality rate of 65.8% in GIDID, with a mean age of death at 11.8 months. Clinical observations indicate that patients with the ID/MIA phenotype tend to have earlier mortality compared to those with the ID/E phenotype (4). Zhaowei Huang et al. conducted a review of GIDID cases caused by TTC7A mutations and found that among the 89 reported patients, the majority had a poor prognosis, with 52.8% deceased and 43.8% surviving. The median age of the deceased and surviving patients was 1.83 years and 0.92 years, respectively, with most deaths occurring before the age of two (16).

Recent advances in genetic research have significantly enhanced our understanding of the molecular basis of GIDID, with a growing body of literature identifying TTC7A mutations as the most prevalent pathogenic variant associated with this condition (6). However, current evidence suggests that the contribution of genetic factors to GIDID pathogenesis remains substantially underestimated. The precise identification of genetic causes plays a pivotal role in clinical management, as it not only facilitates accurate prognosis but also enables reliable estimation of recurrence risks and informs evidence-based therapeutic strategies.

The TTC7A gene, located on chromosome 2p21, comprises 20 exons and encodes a protein of 858 amino acids (17). This gene exhibits widespread expression across multiple tissues and organs, including the brain, bone marrow, testes, pancreas, ovaries, liver, and blood. The encoded TTC7A protein is structurally characterized by the presence of tetratricopeptide repeat (TPR) domains, which are defined by a degenerate consensus sequence of 34 amino acids typically arranged in tandem arrays (18). TPR domain-containing proteins, such as TTC7A, are known to participate in a wide range of critical biological processes, including transcriptional regulation, mitochondrial and peroxisomal protein transport, protein kinase inhibition, cell cycle control, NADPH oxidase activity modulation, and viral replication (19).

Recent studies indicate that TTC7A plays a crucial role in the development and maintenance of intestinal epithelial cells. TTC7A contains nine tetratricopeptide repeat (TPR) domains, which are well-conserved structural motifs frequently observed in proteins involved in cellular trafficking and scaffolding processes (20). Functionally, TTC7A serves as a scaffold protein that facilitates the transport of phosphatidylinositol 4-kinase III alpha (PI4KIIIα) to the plasma membrane. At this site, PI4KIIIα catalyzes the phosphorylation of phosphatidylinositol (PI) to generate phosphatidylinositol 4-phosphate (PI4P) (1). The maintenance of optimal PI4P levels is critical for several cellular processes, including plasma membrane identity establishment, apicobasal polarity formation, cell survival regulation, and the biosynthesis of poly-phosphorylated phosphatidylinositol phosphate lipids such as PI(4,5)P2 and PI(3,4,5)P3. Notably, the synthesis of plasma membrane PI4P is entirely dependent on the proper function and localization of PI4KIIIα (21–23). AKT, a key survival kinase, exerts its protective effects through phosphorylation-mediated activation, which subsequently inhibits apoptosis, promotes cellular proliferation, enhances protein synthesis, and regulates metabolic processes (23). In the context of TTC7A deficiency, studies have demonstrated a marked reduction in phosphorylated AKT (p-AKT) levels within the intestinal epithelium, while total AKT levels remain relatively stable. This observation suggests that impaired AKT activation, as reflected by diminished p-AKT levels, may represent a critical mechanism underlying the pathophysiology of TTC7A deficiency (7).

Research conducted by several scholars has demonstrated that mutations in the TTC7A gene, leading to TTC7A deficiency, disrupt the RhoA signaling pathway (2). Although this disruption does not prevent intestinal development, it partially compromises epithelial cell polarity and intestinal homeostasis. Specifically, TTC7A deficiency results in the abnormal phosphorylation of key ROCK effectors, including ezrin-radixin-moesin (ERM) proteins and myosin light chain (MLC), which are critical regulators of cell adhesion, polarization, and migration (24–26). Furthermore, in T lymphocytes, TTC7A deficiency has been shown to impair their proliferation, adhesion, and migration within the thymus, highlighting the broader systemic impact of TTC7A dysfunction beyond the intestinal epithelium (27).

A research team developed mouse models, including the flaky skin mutant (*fsn*), hereditary erythroblastic anemia (*hea*), and INT mutants (*int*), which revealed spontaneous mutations in the TTC7A gene (28). Pathological analysis of these models demonstrated significant gastric abnormalities, including extensive papillomatosis of the stratified squamous epithelium in the forestomach, elevated apoptosis of cecal enterocytes, and a moderate mixed inflammatory cell infiltrate within the lamina propria. Furthermore, some mice exhibited a hyperproliferative immune disorder characterized by splenomegaly, lymphadenopathy (marked by the accumulation of T and B lymphocytes), and systemic autoimmune features that persisted over time. The structural similarity between mouse and human TTC7A orthologs is notable, with both species sharing 20 exons that encode an 858-amino acid protein containing 7 TPR motifs, resulting in a 96 kDa protein. These findings provide compelling evidence that defects in the TTC7A gene are directly associated with gastrointestinal abnormalities and immune dysregulation (29–32).

## 5 Conclusion

In conclusion, we present a rare case of prenatal diagnostic imaging in GIDID fetuses caused by TTC7A gene mutations, a clinical presentation that has been scarcely documented in the literature. In this case, the observed fetal phenotype is highly consistent with the identified genotype, providing strong clinical evidence supporting the pathogenicity of both variants. Protein structure predictions revealed that the c.2357G>T missense variant causes a significant structural disruption, while c.2378del frameshift mutation alters an amino acid residue. Although the c.2378del frameshift mutation did not result in a marked alteration of the predicted overall protein structure, we infer that the resulting glycine-to-alanine substitution—despite involving two amino acids with similar physicochemical properties—may occur within a functionally critical domain, thereby disrupting protein function. This case not only expands the known clinical phenotype of the disease but also highlights the critical importance of early detection and accurate diagnosis. For fetuses exhibiting gastrointestinal abnormalities and/or immune dysfunction, GIDID should be included in the differential diagnosis, and prompt genetic testing for TTC7A mutations is strongly recommended to facilitate timely clinical management.

## Data Availability

The datasets for this article are not publicly available due to concerns regarding participant/patient anonymity. Requests to access the datasets should be directed to the corresponding author.
